# Factors influencing orthodontic treatment time for non-surgical Class III malocclusion

**DOI:** 10.1590/1678-775720150353

**Published:** 2016

**Authors:** Lívia Monteiro Bichara, Mônica Lídia Castro de Aragón, Gustavo Antônio Martins Brandão, David Normando

**Affiliations:** Universidade Federal do Pará, Faculdade de Odontologia, Belém, PA, Brasil

**Keywords:** Orthodontics treatment, Angle Class III malocclusion, Treatment efficiency

## Abstract

To improve orthodontic treatment efficiency, orthodontists must know which variables could interfere with orthodontic treatment time.

**Objective:**

To identify variables and their effect size on orthodontic treatment time of Class III malocclusion.

**Material and Methods:**

Forty-five Class III malocclusion cases were selected from 2008 patients’ records. Clinical charts, cephalometric radiographs, and pre and posttreatment dental casts were evaluated. Age, sex, PAR index at T1 and T2, overjet, missing teeth, extractions, number of treatment phases, missed appointments, appliance breakages, and cephalometric variables SNA, SNB, ANB, Wits, SnGoGn, CoA, CoGn, IMPA, 1.PP were investigated by multiple linear regression analysis and stepwise method at p<0.05. The sample was also divided into two groups: Group 0-2 (patients who had missed two clinical appointments or less) and Group >2 (patients who missed more than 2 appointments), to detect the influence of this data on treatment time and the quality of the treatment (PAR T2).

**Results:**

Average treatment time was 30.27 months. Multiple regression analysis showed that missed appointment (R2=0.4345) and appliance breakages (R2=0.0596) are the only variables able to significantly predict treatment duration. Treatment time for patients who missed more than 2 appointments was nearly one year longer. However, no significant influence on PAR T2 was observed for those patients.

**Conclusion:**

Orthodontic treatment duration in Class III patients is mainly influenced by factors related to patient compliance. Patients who missed more appointments did not show worse orthodontic finishing, but longer treatment. No occlusal, cephalometric, or demographic variable obtained before treatment was able to give some significant prediction about treatment time in Class III patients.

## INTRODUCTION

Orthodontic treatment duration has always been a major concern to both patients and professionals. In an attempt to predict treatment costs, patients want to know how long their orthodontic treatment will take^6^. Likewise, braces can cause discomfort and inconveniences related to daily routine changes.

For orthodontists, a more precise prediction of the duration of a treatment can earn patients' trust, representing a valuable tool for a successful treatment^26^. “Truth and accurate time estimation” are two of the most frequent recommendations, followed by “reduction in treatment fees”^19^. Also, orthodontic treatment has biological costs and long treatments have been associated with root resorption^17,22^. Therefore, a better understanding of the factors influencing treatment time can provide superior cost-benefit outcomes, as it allows orthodontists to manage treatment, achieving, thus, great results in less time.

Factors that could interfere on treatment duration include sex^11^, pretreatment ANB value^11,27^ overbite^27^, crowding^2,27^, extractions^2,11,27^, time between appointments^2^, treatment phases^29^, age^10,20^, overjet^6,20^, technique^26^, patient compliance (including missed appointments and debonds)^16,20,27^public or private practice^30^, oral hygiene^12,27^, scholar grades^12^, caries^12^, restorations^12^, arch coordination^12^, parent's occupation^12^.

However, to the best of our knowledge, all previous studies, including a systematic review^15^, focus on treatment duration of Class I and II malocclusion subjects. Only association between Class III molar relationship and treatment time has been described^31^. This might be due to the low prevalence of Class III malocclusion, around 5% of a population,^7,18^ and the high acceptance of treatment need by professionals and patients for those cases^7,9,21^.

Class III malocclusion has particular characteristics that differ from others malocclusions. Despite its low prevalence^9,21^, the impact on life-quality is high^7^. Also, Class III growth pattern has some particularities when compared with Class I and Class II patients, as more vertical pattern and longer growth peak for Class III than for Class I patients^4^. While mandibular growth works for the benefit of Class II treatment, in Class III patients’ mandibular growth imposes one more difficulty. Furthermore, relapse after orthodontic treatment is frequently reported^8^.

The knowledge of which variables can interfere in Class III treatment duration might help clinicians to act upon controllable variables, performing more efficient Class III treatment. Therefore, our objective is to evaluate variables present in the orthodontic intervention for Class III malocclusion that could influence treatment duration.

## MATERIAL AND METHODS

This study received ethical approval from The Research Ethics Committee of the Federal University of Pará (number 517.398, 2014). Sample size was estimated using GPower 3.1 software. To detect a 0.35 effect size using six independent variables, alfa level of 0.05, and power of 0.8, we needed to assess 46 patients. Forty-five (19 female and 26male) consecutively treated Class III patients were retrospectively selected from 2008 files of an experienced orthodontist. The inclusion criteria were: non-syndromic dental Class III subjects with Class III molar relationship; edge to edge incisor relationship or anterior crossbite; permanent dentition treated with full orthodontic appliance in both arches. Exclusion criteria comprised patients who had more than one missing tooth *per* hemi arch, who missed over 16 appointments, and those who were surgically treated. No patient had TAD's placed before or during treatment or were treated with self-ligating brackets.

Data were collected from clinical records, dental casts, and cephalometric radiographs. The ages at the beginning of treatment (T_1_) ranged from 9.5 to 48 years old, and mean age was 22.02 years. Treatment was performed using preadjusted twin brackets with .022x.028 slot.

**Figure 1:**
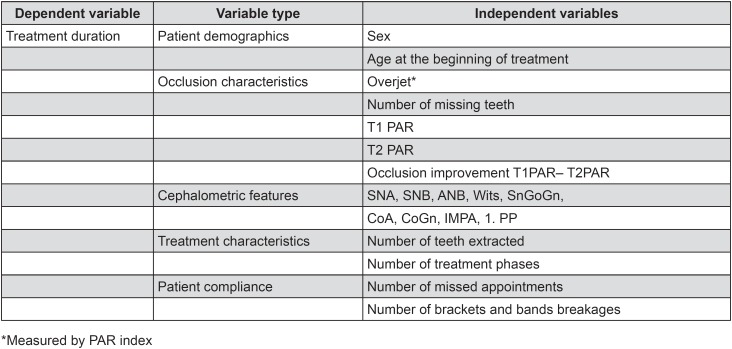
Variables analyzed in the study

The information collected from dental records were age, sex, duration of orthodontic treatment, number of treatment phases, number of teeth extractions due to treatment plan, number of missing teeth before treatment, missed appointments, and appliance breakages (Figure 1). Sagittal incisor relationship was evaluated based on the overjet section of PAR index. Each interval longer than 45 days between two consecutive clinical visits were considered as missed appointment. The number of extractions or missing teeth was sought in the clinical records and confirmed using panoramic radiographs from before and after treatment. Dentoskeletal measurements SNA, SNB, ANB, Wits, SnGoGn, CoA, CoGn, IMPA, and 1.PP were obtained from cephalometric radiographs, aiming to find if treatment time is influenced by cephalometric variables.

Dental casts were assessed to obtain PAR index before (T_1_) and after orthodontic treatment (T_2_), according to Richmond, et al.^23^ (1992), using a digital caliper (Mitutoyo- Suzano, São Paulo, Brazil). PAR index^23^ was doubled measured in twenty dental casts with a 30-day interval. All data retrieved from dental casts and cephalometric radiographs were confirmed by a second examiner. Intra-Class correlation test was calculated to evaluate the reliability of measurements.

Correlation between treatment duration (dependent variable) and continuous variables retrieved from patients' records was analyzed using Pearson's Correlation test. The Student's *t* test for 2 independent samples was applied to search for differences in treatment duration between genders. Then, multiple linear regression was used to examine the influence of independent variables on orthodontic treatment time (dependent variable).

The sample was divided in 2 groups regarding the number of missed appointments to verify if it was related to treatment duration and PAR at T_2_ Patients with 0 to 2 missed appointments were gathered in Group 0-2 (n=27; 18 male, 9 female), and patients with more than 2 missed appointments in Group >2 (n=18; 9 male and 9 female). Normal distribution was verified using D'Agostino-Pearson's test and descriptive statistics were calculated. Student *t* test was applied to evaluate differences between group variables with normal distribution, and, for variables with abnormal distribution, Mann-Whitney test was applied.

Statistical analysis was performed with Bioestat 5.3 software (Mamirauá Institute, Belém, Pará, Brazil). All tests applied used the level of significance at 5%.

## RESULTS

An excellent reliability of PAR index measurements was observed (ICC=0.9541, p<0.001). Mean treatment time for Class III subjects was of 30.27 months (ranging from 11.12 to 54.96).

Patient compliance, featured as the number of appliance breakages (r=0.4195, p=0.004) and missed appointments (r=0.6595, p<0.0001), treatment characteristics PAR at T_1_(r=0.3251, p=0.029), PAR at T_2_ (r=0.349, p=0.03229), and skeletal features SNB (r=-0.3434, p=0.02), SnGoGn (r=0.3532, p=0.017) were found to have significant correlation with orthodontic treatment duration (Table 1). These variables were included in the multiple regression model. Patient's demographics, number of orthodontic treatment phases, overjet, number of missing teeth, number of teeth extracted, treatment improvement (PAR T_1_-T_2_), and the other cephalometric measurements SNA, ANB, Wits, CoA, CoGn, IMPA and 1.PP had no association with treatment duration. Also, no difference was found between male and female concerning treatment time (p=0.41). Therefore, these variables were not included in the multiple regression model.

**Table 1:** Mean, standard deviation (SD), Coefficient of Correlation (r) and p-value of association with treatment duration for each variable assessed in the study

Variables						
	Mean/ Median	SD	Min	Max	r	p-value
Treatment duration	30.27	10.76	10	54.93	–	–
Age at the beginning of treatment	22.2	9.57	9.58	48.75	0.095	0.532
Overjet (PAR index for incisors)	14.60	7.65	0	24	0.159	0.2912
Number of broken brackets or bands	2	3.89	0	16	0.419	0.0041*
Missed appointments	2	3.87	0	16	0.659	<0.0001*
Missing teeth	0	1.32	0	4	0.068	0.6546
Extracted teeth	0	1.21	0	4	-0.044	0.7713
PAR T1	30.28	12.28	3	54	0.3251	0.0292*
PAR T2	2.95	2.17	0	8	0.3229	0.0304*
Occlusion improvement (PAR T1 - PAR T2)	27.3	11.96	2	53	0.177	0.2386
SNA	82.25	4.55	69	93	-0.252	0.0946
SNB	83.09	3.93	73.9	91	-0.343	0.0209*
ANB	-0.95	2.55	-7.5	3.6	0.1152	0.45
Wits	-5.7	2.61	-12	0	-0.036	0.7806
SnGoGn	32.43	5.8	17.9	44.9	0.3532	0.0173*
CoA	92	7.3	74	106	0.0413	0.7878
CoGn	127.4	10.06	104	151	0.0674	0.662
IMPA	83.37	6.47	64.2	94.2	0.0338	0.8257
1.PP	118.51	7.8	97.9	132.4	-0.282	0.0587
	Frequency					
Number of treatment phases	1 Phase 64% 2 Phase 36%				-0.102	0.5040

* Statistical significance p-value ≤0.05

Results after multiple regression linear test and stepwise regression showed that around half of treatment duration (R^2^=0.4944) could be predicted by two variables: missed appointments (R^2^=0.4345, p=0.0002), followed by the number of debonded brackets and bands (R^2^=0.0596, p=0.0241). The cephalometric measurements SnGoGn (R^2^=0.0322) and SNB angle (R^2^= 0.0008), and malocclusion index PAR at T_1_ (R^2^=0.0275) and PAR at T_2_ (R^2^=0.0204) had some influence over treatment duration, though not statistically significant (Table 2). Each missed appointment was found to add about 1.5 months to the treatment duration.

**Table 2:** Multiple linear regression and stepwise statistics of independent variables showing significant correlation with class III treatment duration (F=9.99, <0.0001)

Variables			
		R2	P
Missed appointments	1.38	0.4345	0.0002
Debonded brackets or bands	0.74	0.0596	0.0241
PAR T1	0.17	0.0275	0.1437
PAR T2	0.99	0.0204	0.1399
SNB	-0.26	0.0008	0.8093
SnGoGn	0.09	0.0322	0.7096

There was a statistically significant difference (p=0.02) between Group 0-2 (mean= 27.01±7.56 months) and Group >2 (mean= 36.15±11.29 months) for treatment duration. The PAR index at T_2_ did not show statistically significant difference (p=0.098) when patients with fewer missed clinical visits were compared with those who had more missed appointments.

## DISCUSSION

“How long will my treatment last?” is one of the most common questions asked by patients seeking for orthodontic treatment. To answer it, the orthodontist should focus on which variables could interfere with the treatment progress. Although several studies have investigated factors associated with treatment duration for Class I or Class II malocclusion patients, to the best of our knowledge, just one study^31^ gives some information about duration of Class III malocclusion treatment. This previous study only analyzes the influence of molar Class III position on treatment duration in a small sample. No significant association with treatment duration was found. These data would be also indispensable for future investigations on treatment types and effectiveness of results.

Previous reports evaluating adult patients^16^ showed that the amount of missed appointments is the factor that affects treatment duration (43.75%) of Class I and Class II patients the most. These findings are supported by our findings (43.49%). This is a valuable information for orthodontists, since the patient also assumes some of the responsibility for the treatment time and it can persuade the patient into having good compliance.

Appliance breakages were weak, but statistically associated with treatment time in this study (R^2^=0.0596), as previously described^6,16,20^. Increments in treatment duration might be due to the necessity of returning to a lighter arch wire or the impossibility of treatment evolution in that month.

Our findings indicate that patient cooperation appears to have a greater effect on duration of orthodontic treatment in Class III patients. This might occur since it is known that moderate to severe Class III malocclusions can have a considerable impact on patient's aesthetics and quality of life, keeping them more motivated and easy to handle. Clinically, this motivation should be increasingly utilized toward a shorter treatment duration.

A previous study^1^ reported that missed appointments and appliance repairs explained 30.6% of treatment duration. A different study^20^ also shows that total brackets or bands breakage affects orthodontic treatment duration in teenage patients; however, no significant influence was found by the authors regarding missed appointments. Maybe this could be explained by the fact that adolescents are more likely to accept parent control; therefore more assiduous than older adolescents or young adults. Furthermore, intermaxillary elastics^24^ are quite often required in Class III compensatory treatment, demanding good patient compliance.

Peer Assessment Rating (PAR) was used to quantify the severity of the malocclusion given that it is a valid and reliable method: the higher the index, the greater the amount of malocclusion of the patient. PAR index at T_1_ and T_2_ showed no significant association (p>0.05) with treatment time. A possible explanation for this is the high PAR T_1_, which reflects poor patient compliance. When the requirement of continuously using elastics is not met, the establishment of good occlusal relationship is affected, influencing the final PAR index.

No statistical difference was found among patients who missed zero to two appointments and patients who missed more than two appointments during treatment regarding the final PAR index. The fact that a longer treatment time was necessary in Group >2 to obtain an orthodontic outcome similar to that of group 0-2 indicates that obtaining good occlusal finishing in noncompliant patients requires longer treatment time.

Age had no influence on treatment time in this study at the beginning of Class III treatment, differing from previous investigations examining Class I and Class II malocclusion^6,20,30^. Therefore, other pretreatment or external factors, not included in this study, might be the reason why patients are skipping appointments.

When comparing only fixed treatment length, the literature shows no difference among patients treated for Class II malocclusion in one and two phases^9^ as our findings. However, this does not mean that the first phase of the treatment is unnecessary. Most Class III patients who seek for treatment in a younger age have more severe malocclusion^14,25^. Usually, the first phase includes an orthopedic expansion and maxillary protraction. Consequently, most of the time, second phase involves only a compensatory treatment with fixed appliance.

Differently from our findings concerning Class III malocclusion, some reports on Class II patients describe an association between overjet and treatment duration^12,20^. Initial positioning of upper and lower anterior teeth and mandibular growth are not favorable to non-surgical Class III treatment^13,28^. Frequently, the upper incisors show compensatory protrusion while the lowers have lingual inclinations, limiting the amount of negative overjet that can be treated without surgery. Nevertheless, Class II division 1 patients have proclined upper incisors^25^, which is favorable for compensatory treatment.

Another factor regarding Class III treatment is that severe anterior crossbite is often related with substantial and evident skeletal discrepancies, requiring surgical treatment^3^, unlike Class II patients, which skeletal discrepancies are more “aesthetically acceptable”^1,5^ and can be treated in a compensatory manner.

Treatment involving extractions and missing teeth before treatment had no statistically significant influence on treatment duration. Space closure can be a time-consuming treatment phase^23^; however, extractions can increase treatment efficiency when they are correctly indicated.

This study had some methodological limitations, such as using a retrospectively selected unicenter sample. However, it is a consecutively treated sample, which decreases the risk of bias. Another limitation of our study is the non-inclusion of surgical patients in the sample, leaving out a large number of Class III cases available in the office files. Evaluating this variable, it would have been important to verify the impact of conducting surgical treatment on treatment duration in Class III patients.

Failure to meet the estimated treatment time frequently damages the doctor-patient relationship by decreasing the patient's trust. Biologically, elongated treatment time have been related to increased probability of root resorption^17,22^. Therefore, the awareness of the factors contributing to treatment overtime can help orthodontists to control some of these variables and perform a more efficient treatment for Class III malocclusion, having smoother relationship with patients and greater practice success. Our findings showed that duration of orthodontic treatment in Class III dental malocclusion patients is mainly influenced by patient compliance. Thus, it seems crucial to inform patients about their role in the treatment progress and provide scientifically sound data to stimulate patient's cooperation.

## CONCLUSION

No variable obtained before treatment was able to give some prediction about treatment time in this sample of Class III patients.

Patient cooperation was associated with approximately 50% of the variation in treatment time for Class III patients. Therefore, it seems necessary to seek for strategies that may encourage patient cooperation during orthodontic treatment.

Other variables, such as surgery need, not included in this study, should be investigated.
